# Development of Folic Acid-Conjugated and Methylene Blue-Adsorbed Au@TNA Nanoparticles for Enhanced Photodynamic Therapy of Bladder Cancer Cells

**DOI:** 10.3390/nano10071351

**Published:** 2020-07-10

**Authors:** Che-Wei Hsu, Nai-Chi Cheng, Mei-Yi Liao, Ting-Yu Cheng, Yi-Chun Chiu

**Affiliations:** 1 Division of Urology, Department of Surgery, Taipei City Hospital Zhongxiao Branch, Taipei 115, Taiwan; DAX82@tpech.gov.tw; 2 Department of Applied Chemistry, National University of Kaohsiung, Kaohsiung 811, Taiwan; ferrero565@gmail.com; 3 Department of Applied Chemistry, National Pingtung University, Pingtung 900, Taiwan; myliao@mail.nptu.edu.tw (M.-Y.L.); t0922460558@gmail.com (T.-Y.C.); 4 Division of Urology, Department of Surgery, Taipei City Hospital Heping Fuyou Branch, Taipei 100, Taiwan; 5 Department of Exercise and Health Sciences, University of Taipei, Taipei 100, Taiwan; 6 Department of Urology, School of Medicine, National Yang-Ming University, Taipei 112, Taiwan

**Keywords:** photodynamic therapy, bladder cancer, photosensitizers, Au@TNA nanoparticles, phototoxicity, photomedicine

## Abstract

Photodynamic therapy (PDT) is a promising treatment for malignancy. However, the low molecular solubility of photosensitizers (PSs) with a low accumulation at borderline malignant potential lesions results in the tardy and ineffective management of recurrent urothelial carcinoma. Herein, we used tannic acid (TNA), a green precursor, to reduce HAuCl_4_ in order to generate Au@TNA core-shell nanoparticles. The photosensitizer methylene blue (MB) was subsequently adsorbed onto the surface of the Au@TNA nanoparticles, leading to the incorporation of a PS within the organic shell of the Au nanoparticle nanosupport, denoted as Au@TNA@MB nanoparticles (NPs). By modifying the surface of the Au@TNA@MB NPs with the ligand folate acid (FA) using NH_2_-PEG-NH_2_ as a linker, we demonstrated that the targeted delivery strategy achieved a high accumulation of PSs in cancer cells. The cell viability of T24 cells decreased to 87.1%, 57.1%, and 26.6% upon treatment with 10 ppm_[Au]_ Au@TNA/MB NPs after 45 min, 2 h, and 4 h of incubation, respectively. We also applied the same targeted PDT treatment to normal urothelial SV-HUC-1 cells and observed minor phototoxicity, indicating that this safe photomedicine shows promise for applications aiming to achieve the local depletion of cancer sites without side effects.

## 1. Introduction

Bladder cancer is the 10th most common cancer worldwide, with an estimated 549,000 new cases and 200,000 deaths each year [1]. Nearly 70% of bladder cancer cases are superficial or non-muscle-invasive bladder cancer (NMIBC) at initial presentation [2]. Initial treatment options include complete transurethral resection (TUR), followed by intravesical bacillus Calmette–Guérin (BCG) [3,4]. Nevertheless, 55% of NMIBC patients develop recurrence with limited treatment options, and 20% progress to muscle-invasive bladder cancer (MIBC) within 5 years [5]. The condition of those patients will exacerbate rapidly if left untreated, and the mortality within 2 years is 85% [6]. A multidisciplinary therapeutic approach tailored to individual patients, including surgery, systemic chemotherapy, and radiotherapy, is often required to improve survival; however, these treatments may cause severe adverse effects and affect the quality of life. These management steps will affect the daily urinary storage and voiding function of the bladder, which is crucial for allowing patients to eliminate waste from the body, regulate their blood pressure, and control levels of electrolytes. Focal surgical intervention combined with minimally invasive therapeutic techniques for bladder cancer would alleviate the adverse effects and inconvenience caused by the long-term course of treatment [7].

Photodynamic therapy (PDT) is a minimally invasive therapeutic procedure used for malignant cells that involves the administration of a photosensitizer (PS) followed by irradiation at a wavelength corresponding to the absorbance band of the PS and a series of events leading to direct tumor cell death [8]. The clinical use of many photosensitizers has been hampered by their nonspecific damage to normal tissues, environmental degradation or hydrophobicity, and poor cellular uptake [9]. The exact reasons for the preferential accumulation of PSs in cancer tissue have not been clearly elucidated. Some of the hypotheses include leaky tumor vasculature, reduced lymphatic drainage, a low interstitial pH, and a high number of low-density lipoprotein (LDL) receptors [10]. The most widely discussed photosensitizers are porfimer sodium (Photofrin) and hexaminolevulinate (HAL). The response rate to conventional therapy from combined series is 66% in patients with carcinoma in situ (CIS) of the bladder refractory [11]. Most lipophilic photosensitizers associated with PDT (e.g., Photofrin) localize to the mitochondria and induce apoptosis through mitochondrial disruption, the release of cytochrome c, and activation of the intrinsic pathway of apoptosis [12]. Because of their lipophilicity, they tend to enter both normal and neoplastic cells, leading to collateral damage [13]. The dermal sensitivity and bladder toxicity of PSs not only limit the safe dose, but also induce significant side effects, such as skin photosensitivity, bladder contracture, fibrosis, irritability, and even a loss of bladder capacity. A new targeted PS delivery approach has emerged from the PDT method; this approach combines a very hydrophilic PS to minimize nonspecific accumulation due to the natural hydrophobicity of normal urothelial cells and a PS conjugated to monoclonal antibodies specifically selected for proteins overexpressed on the surface of cancer cells [10]. Although selectively targeting antibody-conjugated photosensitizers to tumors can protect normal urothelium cells and avert adverse events, the amount of drug delivered remains lower than those obtained using designed carriers based on polymerases, micelles, and high surface-to-volume ratio nanoparticles (NPs).

Noncytotoxic materials such as gold, iron oxide, and silica NPs are becoming promising drug carrier platforms for biomedical applications [14,15]. Gold NPs (AuNPs) are emerging as multifunctional agents for cancer therapy and are being investigated as drug carriers, photothermal agents, and radiosensitizers owing to their high biocompatibility and well-defined optical properties [16,17]. Because potential sensitivity to the oxygen environment may cause unpredictable cytotoxicity, congeneric copper (Cu) [18] and silver (Ag) [19] NPs, or even inert platinum (Pt)-based particles [20], are not applicable drug carriers. Methylene blue (MB) is a phenothiazinium photosensitizer due to its high quantum yield of ^1^O_2_ generation under excitation in the therapeutic window (600–900 nm) and has an excellent membrane-permeable ability [21,22]. MB-encapsulated NP-based phototherapy is very encouraging [23,24] as a photomedicine platform [25] because MB can undergo near-infrared (NIR) excitation to reach deeper depths of tissue. However, the self-aggregation of MB in tumor sites leads to a lower quantum yield of singlet oxygen production, thus limiting its practical application in clinical settings [22].

To deliver massive therapeutic molecules, the application and synthesis of gold NPs have been discussed in many previous works [26,27]; such approaches may require organic materials decorated on the surface of the NPs to prevent particle aggregation and facilitate further bioconjugation [28]. However, preparing a surface passivation layer and carrying PS [29,30] requires postsynthesis reactions and utilizes extra chemical reagents. It is possible that the reaction chemicals residing in the NP carrier may cause unknown toxicity when living cells meet the designed Au-based NPs. Herein, we developed a tannic acid (TNA)-assisted reduction of HAuCl_4_ to form Au@TNA core-shell NPs. The synthesis of gold nanoparticles by the assisted reduction of polyphenols is a simple, low-cost, long-term stable, eco-friendly, and green chemistry approach [31]. Huang and co-workers developed an in-situ reduction process of HAuCl_4_ with tannic acid for the direct deposition of Au atoms onto filter paper [32]. The TNA capping layer was capable of the monomer-based adsorption of MB molecules within the TNA organic shell, leading to a high yield of ^1^O_2_ molecules. The TNA/MB passivation layer readily protected the Au NPs against aggregation in PBS solution over 7 days. MTT assays confirmed the low cytotoxicity of the Au@TNA and Au@TNA@MB NPs. Upon conjugation with folic acid molecules [33,34], Au@TNA/MB exhibited a better selectivity to bind the folate receptor on HeLa cervical cancer cells and T24 bladder cancer cells. Therefore, targeted PDT treatment could produce a large number of reactive oxygen species (ROS) to efficiently damage cancer cells upon 650 nm irradiation, while neither dark toxicity nor a nontoxic PDT effect on the SV-HUC-1 normal bladder cell line was observed. Our approach used a targeted PS to deliver PDT and indicates that the promising next-generation development of selective PDT could be combined with photoimmunotherapy [10] for bladder tumor treatment.

## 2. Materials and Methods

### 2.1. Materials

Tannic acid (TNA), the photosensitizer methylene blue (MB), thiazolyl blue tetrazolium bromide (MTT), and *N*-hydroxysuccinimide (NHS) were obtained from Alfa Aesar. Imidazole (98%), *N,N-*dimethyl-4-nitrosoaniline (RNO), folic acid (FA), and ethyl (dimethylaminopropyl) carbodiimide (EDC) were purchased from Sigma-Aldrich (St. Louis, MO, USA). Hydrogen tetrachloroaurate(III) trihydrate (HAuCl_4_·3H_2_O, 99.9%) was obtained from Bio-Tech. Dimethyl sulfoxide (DMSO) was purchased from Scharlau (Sentmenat, Spain). NH_2_-PEG_3500_-NH_2_ and 9,10-anthracenediyl-bis (methylene) dimalonic acid (ABDA) were purchased from JenKem Technology and FLUKA, respectively. These chemicals were used as received. Deionized water (DI water) was used as a solvent to dissolve the hydrophilic chemicals.

### 2.2. Methods

#### 2.2.1. Preparation of Au@MB NPs

HAuCl_4_ solution (4.5 mL, 1.1 mM) was mixed with fresh TNA solution (0.5 mL, 2.5 mM) at room temperature. After 1 h of reaction, the resulting Au@TNA nanoparticles were collected and purified with DI water by a centrifugation/resuspension process more than three times. The final product was dispersed in 5 mL DI water.

MB powder was dissolved in DI water to prepare a 5 mM mother solution. Afterwards, 25 μL MB (5 mM) was added to the above 5 mL colloidal solution of Au@TNA nanoparticles. The mixture solution was aged for 24 h. After a purification treatment with centrifugation at 10,000× g rpm and redispersion with DI water more than three times to remove the excess MB molecules, Au@TNA@MB nanoparticles were collected. The amount of MB adsorbed onto the surface of Au@TNA nanoparticles was calculated from the difference between the initial optical density of MB and each supernatant collected from centrifugation.

To measure the amount of MB adsorbed onto the Au@TNA nanoparticles, the absorption peak at 660 nm was recorded using a UV-visible spectrometer. The difference between the initial amount of MB and the residue in the supernatant of the Au@TNA and MB mixture solution was calculated, resulting in a 70% reduction ratio. Based on the absorption measurement, 3.4 µM MB was attached to 40 ppm Au@TNA nanoparticles, which corresponded to 27.5 mg _[MB]_/g _[Au]_.

#### 2.2.2. Singlet Oxygen Yielded by Au@TNA@MB NPs and MB

A total of 200 uL Au@TNA@MB (25 ppm_[Au]_) was mixed with 10 uL RNO (1 mM) and 10 µL imidazole (1 mM) solution, and then exposed to 650 nm laser light. The absorption band at 440 nm was recorded as a function of the irradiation time. The same experiment was carried out for assessing the yield of ^1^O_2_ by Au@TNA@MB (25 ppm_[Au]_) and MB solution (0.024 mM).

ABDA was used as another ^1^O_2_ indicator. A total of 200 µL Au@TNA@MB NPs (25 ppm_[Au]_) was mixed with 10 µL ABDA solution (5 mM), followed by the same irradiation process. The absorption change at 400 nm of all samples was recorded.

#### 2.2.3. Synthesis of FA-Conjugated Au@TNA@MB NPs

Two hundred microliters of Au@TNA/MB NP solution (200 ppm_[Au]_) was mixed with 200 μL of NH_2_-PEG-NH_2_ polymer solution (2.8 mg/mL) at 4 °C for one hour, referred to as solution A. Then, 7 mg/mL *N*-(3-dimethyl aminopropyl)-*N*′-ethyl carbodiimide hydrochloride (EDC) and 7 mg/mL *N-*hydroxysuccinimide (NHS) were dissolved in 0.85 mL of FA solution (0.025) to activate the carboxylate groups, referred to as solution B. Solutions A and B were subsequently reacted for another 2 h to form FA-conjugated Au@TNA@MB NPs, where the primary amine groups of the NH_2_-PEG-NH_2_ polymer were used to link the Au@TNA@MB NPs and FA. Centrifugation at 10,000× g rpm and redispersion with DI water more than three times were implemented to purify FA-conjugated Au@TNA@MB NPs.

#### 2.2.4. Cytotoxicity of Au@TNA/MB NPs against Bladder Cancer Cells

A cell toxicity test using a series of as-prepared Au@TNA NPs, Au@TNA@MB NPs, FA-conjugated Au@TNA@MB NPs, Au@TNA@MB NPs plus 660 nm light, and FA-conjugated Au@TNA@MB NPs plus 660 nm light was performed on HeLa cells and T24 cells. HeLa cells, T24 cells, and SV-HUC-1 cells were precultured in DMEM-HG culture medium, McCoy’s 5A medium, and Ham’s F-12K (Kaighn’s) medium, respectively, for 24 h at 37 °C under 5% CO_2_ culture conditions. We tested the cell viability of different cells with Au-based nanoparticles at a density of 8000 cells per well in a 96-well plate. After 1 day of seeding, we dispersed these Au-based nanomaterials in culture media and then added 100 µL (0–200 ppm_[Au]_) to each well to replace the original culture medium. After 45 min, 2 h, and 4 h of coculture, the particle-including medium was removed and then replaced by 100 μL of fresh culture medium. These experimental groups were irradiated with a 660 nm laser (125 mW/cm^2^) for 8 min. The PDT-treated cells or dark-treated (without light) groups were then kept in the incubator for another day. The cell viability was determined by utilizing the MTT assay to measure the ratio difference between the treated groups and the cell-only group (without treatment in the form of particles and laser light).

#### 2.2.5. Cellular Response to Membrane Destruction and Reactive Oxygen Species Detection with Trypan Blue and DCFH-DA

To assess whether the PDT resulted in killing efficiency, living cells treated with Au@TNA@MB NPs plus 660 nm light and FA-conjugated Au@TNA@MB NPs were stained with trypan blue to test the cell viability and with DCFH-DA to analyze the ROS level.

We incubated HeLa cells with 100 μL of the vital stain trypan blue (5 mM) for 30 min after performing PDT treatment (Au@TNA@MB (10 ppm_[Au]_)). PBS solution was added to replace the original staining solution to remove the excess trypan blue. After another 4 h of incubation, a microscope was used to visualize the color pattern of the HeLa cells.

Ten microliters of DCFH-DA stock solution (1 mM) was mixed with 100 μL of PBS solution for the following experiment. One milliliter of T24 cells (10,000 cells/mL) was cultured for 24 h at 37 °C under 5% CO_2_ on a gelatin-coated microscope slide. The medium was removed and replaced with 200 μL of culture medium, including 20 μL of diluted DCFH-DA solution. After an incubation time of 30 min, we used PBS solution to remove the excess DCFH-DA, and 250 μL of Au-based nanomaterials was then loaded into the DCFH-DA stained cells for 4 h. The excess Au-based nanomaterials were removed and the PDT treatment was applied (660 nm laser irradiation at 125 mW/cm^2^ for 8 min). The ROS generation in cell images was investigated by using an Olympus microscope.

### 2.3. Characterization

Transmission electron microscopy (TEM) images were taken by using an H-7500 instrument (HITACHI, Tokyo Japan). High-resolution TEM (HR-TEM) images were recorded by a JEM-2100F (JEOL Ltd., Tokyo, Japan). Powder X-ray diffraction (XRD) analysis was carried out on a Rigaku D/MAX2500 instrument. Ultraviolet-visible spectra were recorded using a V-630 spectroscope (JASCO, Tokyo, Japan). X-ray photoelectron spectroscopy (XPS) analysis was performed on an Ulvac-Phi 5000 Versaprobe instrument (Thermo-VG Scientific, West Sussex, UK). Micro-Raman spectroscopy was performed with a 785 nm laser (DPSSL Driver II, 3.4 mW), an He-Ne laser at 632.8 nm (20 mW), and an MRS-iHR320 modular Raman system, which were integrated into an Olympus BX53 microscope for cellular examinations. The microscope was incorporated with a 20X (N.A. = 0.5) objective lens for SERS sample measurement and combined with Olympus bandpass filter cubes (U-FGW (575–800 nm) and U-FBWA (510–550 nm)) to obtain the red and green emission, respectively.

## 3. Results and Discussion

### 3.1. Characterization of Au@TNA/MB NPs

**Figure 1:**
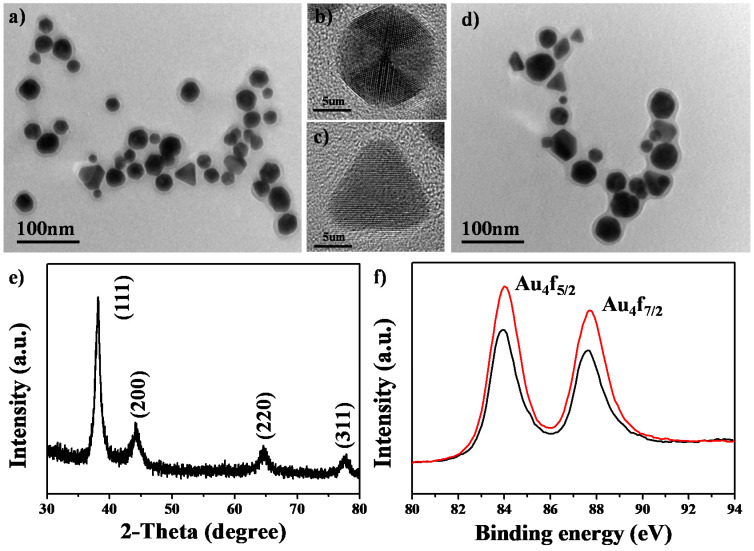
(**a**) Transmission electron microscopy (TEM) image and (**b**,**c**) High-resolution TEM (HR-TEM) images of Au@TNA nanoparticles (NPs). (**d**) TEM image of Au@TNA@MB NPs. (**e**) X-ray diffraction (XRD) pattern of Au@TNA@MB NPs. (**f**) X-ray photoelectron spectroscopy (XPS) gold (Au) 4f orbitals of Au@TNA (red) and Au@TNA@MB (black) NPs.

Figure 1a shows a TEM image of Au@TNA NPs prepared by a one-pot reaction of HAuCl_4_ and TNA solutions. These particles consisted of spherical and triangular shapes with an average particle size of 26.43 ± 3.7 nm (Figure S1). Figure 1b,c shows HR-TEM images of individual Au@TNA NPs with a highly crystalline structure and spherical and triangular shapes, respectively. The crystal lattice space of the periodic packing fringes was 2.35 Å, which was consistent with the (111) diffraction corresponding to face-centered cubic-structured gold. Notably, the spherical gold NP contained three-fold symmetry projections, suggesting the formation of an icosahedral building block structure [35]. Upon the adsorption of methylene blue, the size (26.88 ± 4.6 nm) and shape of the Au@TNA@MB NPs were almost the same as those of the Au@TNA NPs according to the TEM images (Figure 1d). The XRD pattern (Figure 1e) confirmed that the reflection peaks of the Au@TNA@MB NPs corresponded to the face-centered cubic pattern of gold crystallite. Figure 1f shows that the peak positions of Au 4f_5/2_ at 84.0 eV and Au 4f_7/2_ at 87.6 eV were consistent before and after loading MB. The lack of an upshift change at the Au 4f orbital position [36] indicated that the protective TNA interlayer separated sulfur atoms in MB and Au NPs.

**Figure 2:**
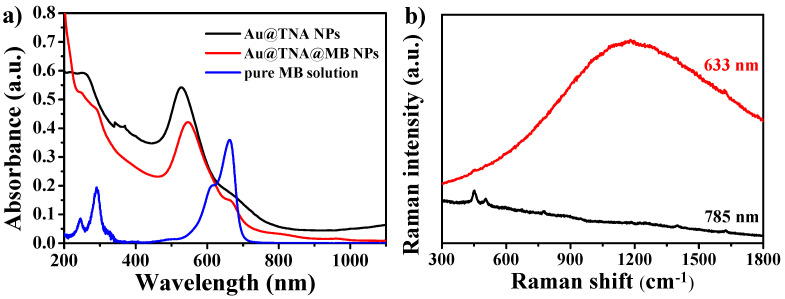
(**a**) UV-visible and (**b**) fluorescence spectra of Au@TNA NPs, Au@TNA@MB NPs, and methylene blue (MB) molecules. (**b**) SERS measurement of Au@TNA@MB NPs at 633 and 785 nm.

Ultraviolet-visible (UV) and fluorescence spectra of Au@TNA and Au@MB NPs from wavelengths between 200 and 900 nm were recorded and are shown in Figure 2a. The absorption peak of the localized surface plasmon resonance (LSPR) of Au@TNA NPs shifted from 528 to 546 nm after the loading of MB molecules. This primary peak moved to a longer wavelength, which could be attributed to the increase in the refractive index after the decoration of a large amount of MB over the surface of Au@TNA NPs. Nearly 70% of MB was adsorbed onto the surface of Au@TNA NPs (Figure S2). Accordingly, we observed that the solution color of Au@TNA NPs changed to a blue-like color for the Au@TNA@MB NP solution (Figure S3). When the Au-based colloids were added to PBS solution, the resulting Au@TNA@MB NP solution showed a high stability against aggregation after 7 days of aging (Figure S3b). In addition, we observed that the maximum absorption peak of MB adsorbed onto the surface of Au@TNA NPs was located at 664 nm, which was slightly redshifted compared with the 662 nm absorption peak of the pure MB solution (Figure 2a). Notably, the absorption band positions of Au@TNA NP and MB were overlapping at 600–650 nm. Additional simulations and experiments are required to examine the potential formation of J- or H-aggregation adsorption [37] of MB molecules on the surface of TNA-decorated Au NPs via hydrogen bonds and/or π–π stacking interactions. Strong fluorescence appeared and negligible surface-enhanced Raman scattering peaks were obtained in the Raman spectrum under laser excitation at 633 nm (Figure 2b). The appearance of fluorescence interference can be attributed to the slight release of MB (about 2.4%) from the Au@TNA@MB NPs (Figure S4). To eliminate the typical fluorescence interference in the SERS spectra from the 633-excited MB molecules, we utilized a 785 nm Raman system to analyze the same solution. The SERS effect of Au@TNA@MB NPs is very weak at 785 nm. These opposite optical phenomena of high fluorescence and low SERS confirmed the existence of the TNA organic shell as a spacer, preventing the close adsorption of MB on the surface of Au NPs.

**Figure 3:**
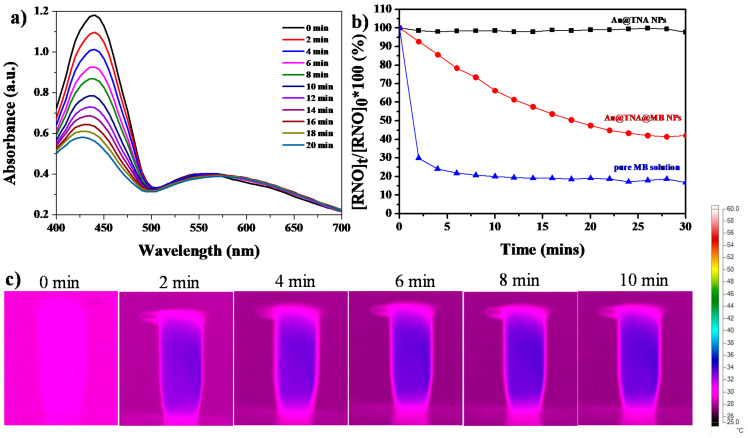
UV-visible spectra (**a**) and relative intensity at 440 nm (**b**) of the *N,N-*dimethyl-4-nitrosoaniline (RNO)/imidazole indicator for measuring single oxygen generation from Au@TNA@MB NPs as a function of the irradiation time at 650 nm. Pure MB and Au@TNA are presented as control groups. (**c**) Thermographic images mapping the temperature increase of 25 ppm_[Au]_ Au@TNA@MB NPs at 650 nm (125 mW/cm^2^).

Next, we detected the production of ^1^O_2_ from Au@TNA@MB NPs (350 ppm_[Au]_) under irradiation with a 660 nm laser. We used a chemical mixture of imidazole and RNO as an indicator to detect the ^1^O_2_ generation of Au@TNA/MB NPs. After reacting Au@TNA/MB NPs and the indicator solution under a 660 nm NIR laser (125 mW/cm^2^), a continuous decrease in the absorption peak at 440 nm was observed from 2 to 16 min (Figure 3a). The relative decreases observed for MB alone, Au@TNA@MB NPs, and Au@TNA NPs were 83%, 42.15%, and 3.2%, respectively (Figure 3b). A similar decreasing trend was observed by using an ABDA indicator. Indeed, Au@TNA@MB NPs appeared to have a lower ^1^O_2_ efficiency when compared with the MB-alone group (Figure S5). When the excited-state MB molecule was transformed and adsorbed on the surface of the Au NPs, the energy quenching process gently decreased ^1^O_2_ generation.

In addition, the Au@TNA@MB NPs exhibited a consistent heating temperature below 38 °C after 10 min of irradiation at 660 nm (Figure 3c). Compared to the MB-alone group (Figure S6), the absorption of Au@TNA@MB NPs showed a negligible change at 664 nm (Figure 3a), which indicated a better photostability of Au@TNA@MB than MB alone. Although pure MB has a higher quantum yield of ^1^O_2_ molecules, it is less applicable than Au@TNA/MB NPs for clinical use because of its unstable properties under prolonged excitation or upon accumulation in tumor sites [22]. On the other hand, the thermographic mapping results show the lack of a significant temperature increase after 8 min of excitation in Figure 3c. This temperature dependence of the Au@TNA/MB NPs (25 ppm) as a function of the laser irradiation time demonstrated a low potential risk of thermal ablation to living cells during 650 nm laser irradiation in the subsequent in vitro studies.

### 3.2. Cytotoxicity of Au@TNA/MB NPs in PDT

**Figure 4:**
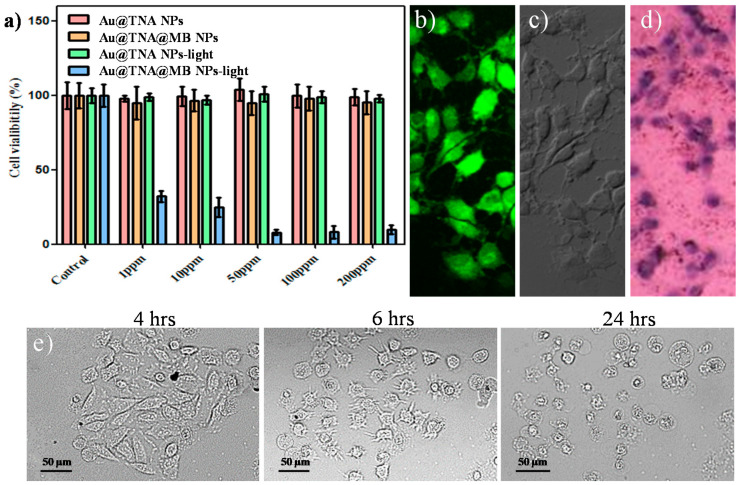
(**a**) Thiazolyl blue tetrazolium bromide (MTT) assay of HeLa cells cotreated with particles for 24 h: Au@TNA, Au@TNA@MB, Au@TNA plus 650 nm light, and Au@TNA@MB plus 650 nm light. (**b**) 2’,7’-dichlorofluorescin diacetate (DCFHDA)-stained HeLa cells, (**c**) bright field image of HeLa cells, and (**d**) trypan blue-stained HeLa cells. The cells were stained after 4 h of treatment with Au@TNA@MB and then exposed to 650 nm light. (**e**) Bright field images of HeLa cell apoptosis after pretreatment with 10 ppm_[Au]_ Au@TNA@MB (4 h) plus 650 nm light. The 650 nm laser power density was 125 mW/cm^2^.

To validate the biocompatibility of Au@TNA/MB NPs, HeLa cells, representing a human cell line derived from a cervical cancer line, were cocultured with solid NPs for 24 h and analyzed by using the MTT assay, in order to evaluate the cell viability (Figure 4a). HeLa cells are one of the most typical cell models used for the preliminary study of PDT treatment models [38,39,40]. As shown in Figure 4a, no dark toxicity towards HeLa cells without light exposure was displayed by 0–200 ppm_[Au]_ Au@TNA@MB NPs. As expected, we observed that 50–200 ppm_[Au]_ Au@TNA@MB NPs effectively reduced the cell viability of HeLa cells to nearly 10% under 660 nm light irradiation. At low sample doses between 1 and 10 ppm_[Au]_, the PDT treatment was mildly harmful, with up to 65% photoinduced cytotoxicity. In contrast, the treatment of Au@TNA NPs plus light was PDT inactive in causing injury to HeLa cells.

The Au@TNA@MB NP-mediated PDT was investigated by using a nonfluorescent compound (2’,7’-dichlorofluorescin diacetate, DCFDA) to produce a green fluorescent molecule (2’,7’-dichlorofluorescein, DCF) when the cells received intracellular reactive oxygen species (ROS). After incubation with Au@TNA@MB NPs (10 ppm_[Au]_) for 4 h at 37 °C under a dark condition, particle-treated HeLa cells were exposed to 650 nm laser light (125 mW/cm^2^) for 8 min. Consequently, the green fluorescence of DCF was clearly observed in Figure 4b for Au@MB NP-treated HeLa cells after light irradiation, being well-matched with the cell location in the bright field image (Figure 4c), indicating the remotely controlled intracellular release of toxic ROS species. In addition, a trypan blue staining method was further used to examine the cell viability of irradiated HeLa cells after 4 h of PDT treatment (Figure 4d). The PDT-treated cells presented a blue color due to the diffusion of trypan blue across the membrane and into the cytoplasm, indicating that the large amount of ROS production within HeLa cells causes cell membrane damage and then triggers cell death. In contrast, the Au@TNA@MB NP w/o light group presented color-free cells, corresponding to a complete cell membrane structure, and thus resisted the inward diffusion of trypan blue. The time-dependent imaging results of PDT treatment are shown in Figure 4e. Consistently, the PDT-treated HeLa cells showed a morphological change: the cell membranes were gradually converted into shriveled and spiked structures from 4 to 6 h, and membrane blebbing appeared at 24 h, possibly through an apoptosis pathway [41]. The apoptotic membrane protrusions of the HeLa cells became round and started to detach from the culture dishes after 24 h of culture. Indeed, the cell volume contraction of PDT-treated HeLa cells from 4 to 24 h, which was at least two-fold, suggests apoptosis pathways [42].

**Figure 5:**
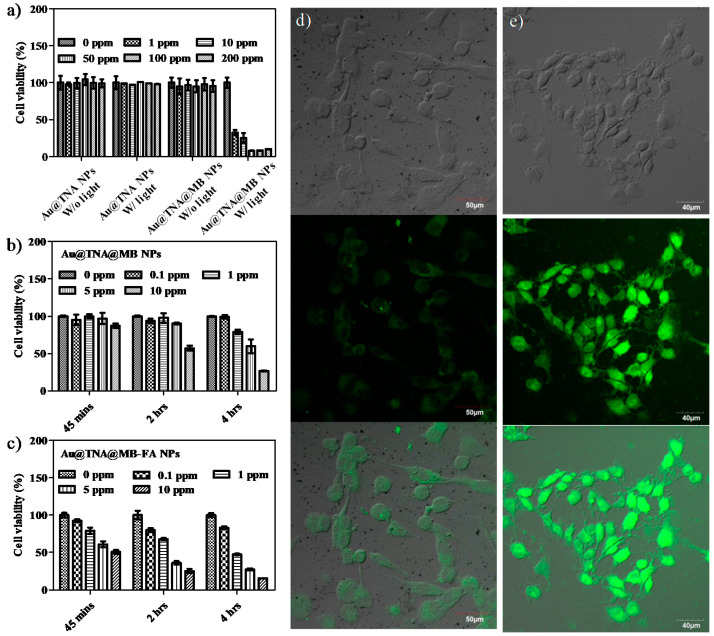
MTT assays of (**a**) T24 cells cotreated with particles for 24 h: Au@TNA, Au@TNA@MB, Au@TNA plus 650 nm light, and Au@TNA@MB plus 650 nm light. T24 cells cotreated with (**b**) Au@TNA@MB NPs and (**c**) folic acid (FA)-conjugated Au@TNA@MB NPs for 0.75–4 h upon excitation at 650 nm. Photodynamic therapy (PDT) treatment of T24 cells stained with DCFH-DA dye by using (**d**) Au@TNA@MB NPs and (**e**) FA-conjugated Au@TNA@MB NPs. Top: bright field image, middle: fluorescent image, and bottom: merged image. The 650 nm laser power density was 125 mW/cm^2^.

Again, we investigated the PDT killing effect on the T24 human bladder cancer cell line incubated with various concentrations (0–200 ppm_[Au]_) of Au@TNA@MB NPs (Figure 5a). A 24 h coincubation followed by PDT was performed. No cytotoxicity against T24 cells was observed when the culture conditions involved Au@TNA NPs, Au@TNA NPs plus light, and Au@TNA@MB NPs alone. The PDT-treated T24 cell group exhibited a remarkably low cell viability at 50–200ppm_[Au]_ and an at least 70% cell death ratio at 1–10 ppm_[Au]_. Figure 5b presents the results of time-dependent and dose-dependent coincubation conditions, which were used to assess the optimal PDT treatment for eliminating bladder cancers at a low drug dose within an ideal reaction time. After 45 min of incubation, 0.1–10 ppm Au@TNA/MB NPs did not have a significant phototoxic effect on T24 cells. There was only a mild killing rate of 42.8% at 10 ppm_[Au]_ Au@TNA/MB NPs for the PDT-treated T24 cells when the incubation time was prolonged to 2 h. At 4 h of incubation, the T24 cell viability with 1, 5, and 10 ppm_[Au]_ Au@TNA/MB NPs decreased in a dose-dependent manner to 78.7%, 59.6%, and 26.6%, respectively.

To achieve active delivery, folic acid (FA) is one of the popular ligands used to bind cancer cells [43]. It has been reported that the number of folate-specific receptor antigens on cancer cells is higher than that for normal cells. Therefore, Au@TNA@MB NPs modified with FA molecules were prepared to evaluate the enhanced PDT treatment of T24 cells. The TEM image in Figure S7a presents the well dispersion for the FA-conjugated Au@TNA@MB NPs. The UV-visible spectrum of FA-conjugated Au@TNA@MB NPs determined a specific absorption pattern (Figure S7b), which is similar to the absorption band in Figure S3b, providing direct evidence for the lack of colloidal aggregation. A slight increase of the zeta potential value from −33.7 mV by Au@TNA@MB NPs to −28.2 mV by FA-conjugated Au@TNA@MB NPs (Figure S7c) was attributed to immobilization of the NH_2_-PEG-NH_2_ polymer and FA molecule. Figure 5c shows that 10 ppm of FA-conjugated Au@TNA@MB NPs was capable of conducting the PDT-triggered killing of at least 50% T24 cells at a 45 min coculture time. When the incubation time was prolonged to 4 h, this enhanced delivery to the specific antigen of T24 cancer cells led to excellent PDT of bladder cancer cells, boosting the death rate to below 50% at 1 ppm_[Au]_ and over 80% at 10 ppm_[Au]_. This high cancer cell mortality could be attributed to the large yield of ROS in the targeted cancer area, according to the comparison of the fluorescence intensity between the targeted PDT results (Figure 5e) and the passive delivery of the targeted-free group (Figure 5d).

**Figure 6:**
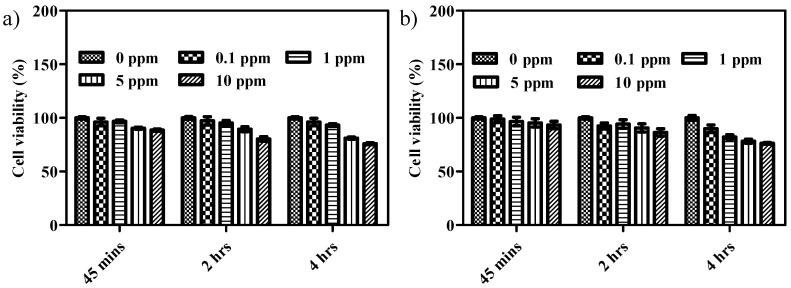
MTT assays of SV-HUC-1 cells cotreated with (**a**) Au@TNA@MB and (**b**) FA-conjugated Au@TNA@MB NPs for 0.75–4 h plus 650 nm light (125 mW/cm^2^), followed by another 24 h of incubation in fresh medium.

Although the enhanced phototoxic effect of FA-conjugated Au@TNA@MB NPs on malignant cells is proven in Figure 5c,e, the potential side effects, e.g., injury to normal cells, remain to be considered. Regarding this, the cell viability of the human normal urinary epithelial cell line SV-HUC-1 was examined under 45-min to 4-h targeted PDT treatments with FA-conjugated Au@TNA@MB NPs. As shown in Figure 6a,b, a very low cytotoxicity for the particle-treated SV-HUC-1 cells (13%) was observed at 10 ppm of FA-conjugated Au@TNA@MB NPs.

## 4. Conclusions

We successfully synthesized Au@TNA NPs by a simple one-pot green reaction of HAuCl4 and TNA solutions without the use of toxic additives. The Au@TNA NPs could carry massive amounts of MB molecules featuring a high production yield of ^1^O_2_ generation upon excitation at 650 nm. The cytotoxicity of Au@TNA/MB NPs was very low for HeLa and T24 living cells at concentrations between 1 and 200 ppm. According to the PDT study with FA-conjugated Au@TNA/MB NPs and the T24 cell line, the target activated photocytotoxicity was better than the passive PDT effect via the endocytosis pathway. The prolonged targeted delivery time to cells could enhance the PDT-related cytotoxicity to cancer cells, but retain a very low dark toxicity to normal cells. Our in vitro study provides promising results for FA-conjugated Au@TNA/MB NPs as prospective PS nanomaterials for PDT to treat NMIBC; nevertheless, additional animal studies are required to prove the treatment efficacy toward cancer cells and to prevent adverse effects. Although it remains a big synthetic challenge to manipulate Au nanostructures with polyphenols, we expect that changing the size and shape of Au@TNA nanoparticles will enhance the affinity to cancer cells and offer a promising future design to improve the delivery efficiency of PDT NPs.

## Supplementary Materials

The following are available online at https://www.mdpi.com/2079-4991/10/7/1351/s1: Figure S1: Size distribution measurements of (a) Au@TNA NPs and (b) Au@TNA@MB NPs on the basis of TEM image measurements; Figure S2: UV-visible spectra of the MB alone and the 1st/3rd supernatant solution collected from the mixture solution of Au@TNA nanoparticle and MB molecule through a centrifugation process; Figure S3: UV-visible measurements of (a) Au@TNA NPs and (b) Au@TNA@MB NPs aged in PBS solution. Optical images of (c) Au@TNA NPs and (d) Au@TNA@MB NPs dispersed in PBS solution for 30 min; Figure S4: (a) UV-visible and (b) fluorescence measurements of the MB alone solution, referred as 100 % of concentration, and the supernatant of the Au@TNA@MB nanoparticles after 1 day of aging; Figure S5: Uv-visible spectra of ABDA reacted with 200 μL of (a) Au@TNA@MB nanoparticles (200 ppm[Au]) and (b) MB alone solution (0.024 mM) under an excitation at 650 nm. The absorption changes at 400 nm of MB alone, Au@TNA NPs, and Au@TNA@MB NPs under an excitation at 650 nm; Figure S6: UV-visible spectra of the RNO/imidazole indicator for measuring single oxygen generation from MB-alone solution as a function of the irradiation time at 650 nm (125 mW/cm^2^); Figure S7: (a) TEM image and (b) UV-visible spectrum of FA-conjugated Au@TNA@MB NPs. (c) Zeta potential measurements of Au@TNA, Au@TNA@MB, and FA-conjugated Au@TNA@MB NPs.
