# Correction: Hsu et al. Development of Folic Acid-Conjugated and Methylene Blue-Adsorbed Au@TNA Nanoparticles for Enhanced Photodynamic Therapy of Bladder Cancer Cells. *Nanomaterials* 2020, *10*, 1351

**DOI:** 10.3390/nano13152233

**Published:** 2023-08-02

**Authors:** Che-Wei Hsu, Nai-Chi Cheng, Mei-Yi Liao, Ting-Yu Cheng, Yi-Chun Chiu

**Affiliations:** 1 Division of Urology, Department of Surgery, Taipei City Hospital Zhongxiao Branch, Taipei 115, Taiwan; 2 Department of Applied Chemistry, National University of Kaohsiung, Kaohsiung 811, Taiwan; 3 Department of Applied Chemistry, National Pingtung University, Pingtung 900, Taiwan; 4 Division of Urology, Department of Surgery, Taipei City Hospital Heping Fuyou Branch, Taipei 100, Taiwan; 5 Department of Exercise and Health Sciences, University of Taipei, Taipei 100, Taiwan; 6 Department of Urology, School of Medicine, National Yang-Ming University, Taipei 112, Taiwan

## Error in Figure

Within the publication of this article in *Nanomaterials* **2020**, *10*, 1351 [1], the authors identified mistakes in the overlap of the images in Figures 4c and 5e, which were possibly made during image collection. Figure 4c is the correct result, and the raw data of Figure 5e is missing. We repeat the same in vitro experiments of the T24 cells treated with (d) Au@TNA@MB NPs and (e) FA-conjugated Au@TNA@MB NPs by staining with DCFHDA with a fluorescence microscope (Olympus IX73). The new results are used to replace the original incorrect data. Corrections to these figures do not affect the results and conclusions of the article. All authors apologize for this and agree to these corrections.

**Figure 5e before correction (Figure 5e was overlapped with Figure 4c).**

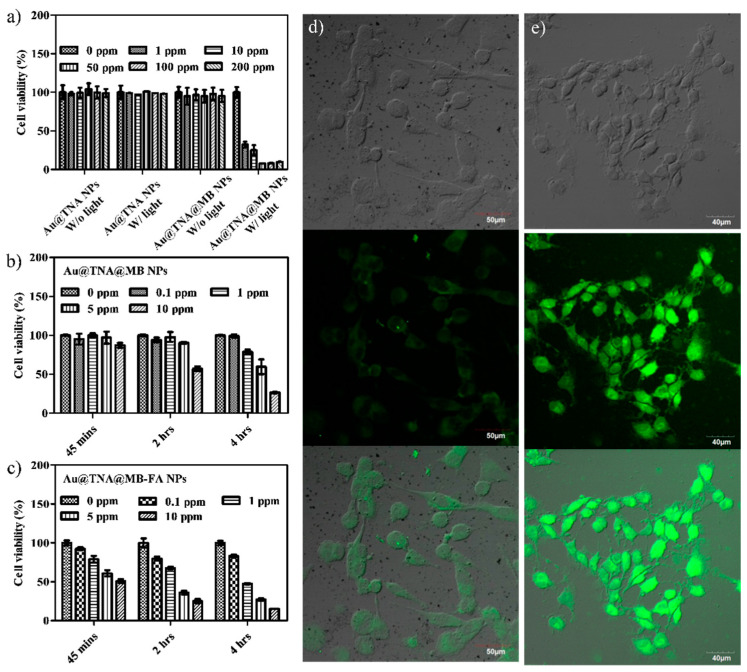



**Corrected Figure 5e and the corresponding change of Figure 5d:**

The authors state that the scientific conclusions are unaffected. This correction was approved by the Academic Editor. The original publication has also been updated.

## Figures and Tables

**Figure 5:**
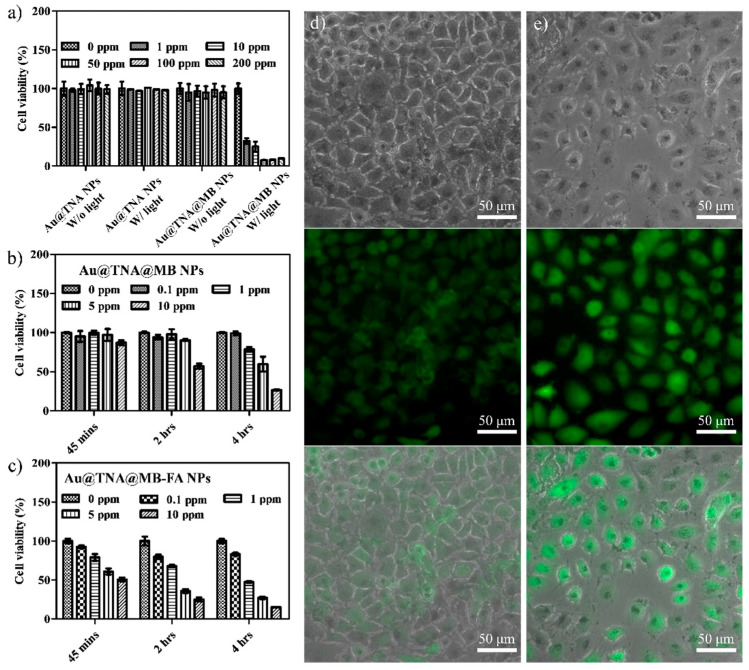
MTT assays of (**a**) T24 cells cotreated with particles for 24 h: Au@TNA, Au@TNA@MB, Au@TNA plus 650 nm light, and Au@TNA@MB plus 650 nm light. T24 cells cotreated with (**b**) Au@TNA@MB NPs and (**c**) folic acid (FA)-conjugated Au@TNA@MB NPs for 0.75–4 h upon excitation at 650 nm. Photodynamic therapy (PDT) treatment of T24 cells stained with DCFH-DA dye by using (**d**) Au@TNA@MB NPs and (**e**) FA-conjugated Au@TNA@MB NPs. Top: bright field image, middle: fluorescent image, and bottom: merged image. The 650 nm laser power density was 125 mW/cm^2^.
