# Supplementary material for: Development of Folic Acid-Conjugated and Methylene Blue-Adsorbed Au@TNA Nanoparticles for Enhanced Photodynamic Therapy of Bladder Cancer Cells
Source: Nanomaterials (Basel). 2020 Jul 10;10(7):1351. doi: 10.3390/nano10071351 (PMC7407911; doi:10.3390/nano10071351)
Supplement: Supplementary file 1 [file nanomaterials-10-01351-s001.pdf]

Supplementary Materials

Development of Folic Acid-Conjugated and Methylene Blue-Adsorbed Au@TNA Nanoparticles for Enhanced Photodynamic Therapy of Bladder Cancer Cells

Che-Wei Hsu [1,†], Nai-Chi Cheng [2,†], Mei-Yi Liao [3,†], Ting-Yu Cheng [3] and Yi-Chun Chiu [4,5,6,*]

[1] Division of Urology, Department of Surgery, Taipei City Hospital Zhongxiao Branch, Taipei 115, Taiwan; DAX82@tpech.gov.tw

[2] Department of Applied Chemistry, National University of Kaohsiung, Kaohsiung 811, Taiwan; ferrero565@gmail.com

[3] Department of Applied Chemistry, National Pingtung University, Pingtung 900, Taiwan; myliao@mail.nptu.edu.tw (M.-Y.L.); t0922460558@gmail.com (T.-Y.C.)

[4] Division of Urology, Department of Surgery, Taipei City Hospital Heping Fuyou Branch, Taipei 100, Taiwan

[5] Department of Exercise and Health Sciences, University of Taipei, Taipei 100, Taiwan

[6] Department of Urology, School of Medicine, National Yang-Ming University, Taipei 112, Taiwan

* Correspondence: DAM15@tpech.gov.tw; Tel.: +886-2-23889595 (ext. #7999)

† These authors contributed equally to this work.

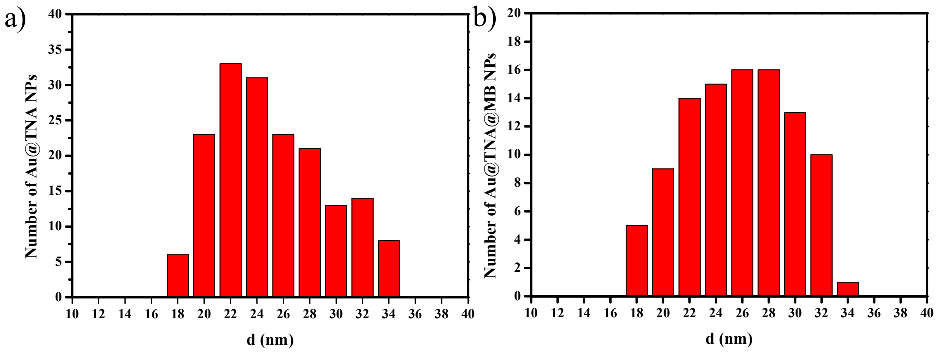

Figure S1 Size distribution measurements of a) Au@TNA NPs and b) Au@TNA@MB NPs on the basis of TEM image measurements.

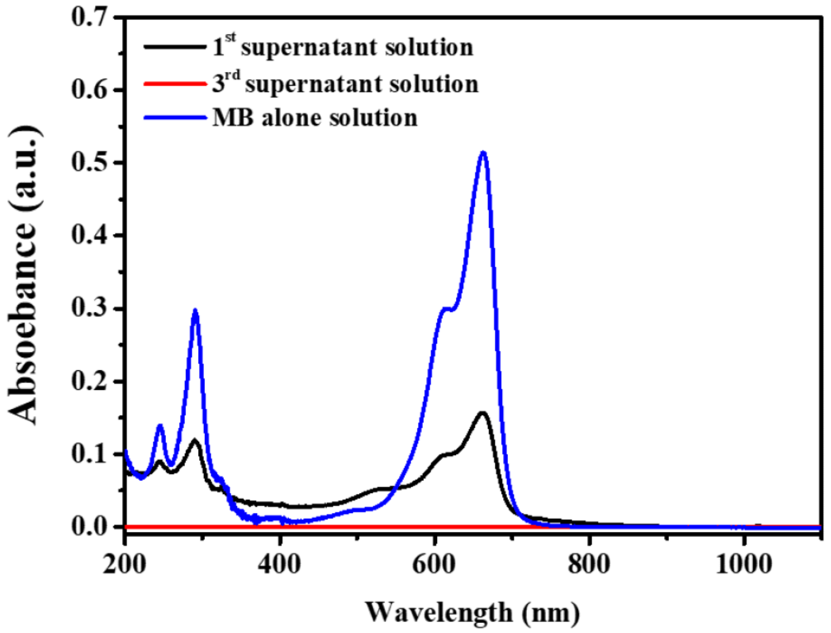

Figure S2. UV-visible spectra of the MB alone and the 1st/3rd supernatant solution collected from the mixture solution of Au@TNA nanoparticle and MB molecule through a centrifugation process.

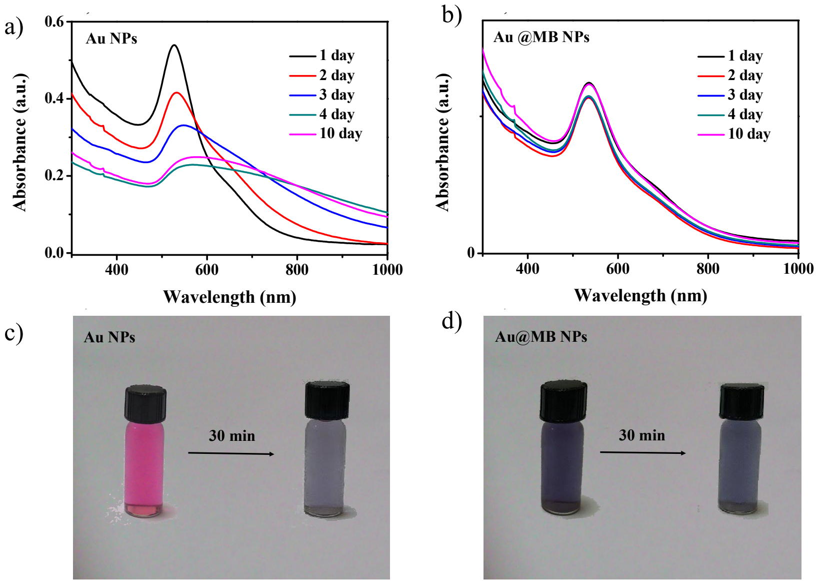

Figure S3. UV-visible measurements of a) Au@TNA NPs and b) Au@TNA@MB NPs aged in PBS solution. Optical images of c) Au@TNA NPs and d) Au@TNA@MB NPs dispersed in PBS solution for 30 min.

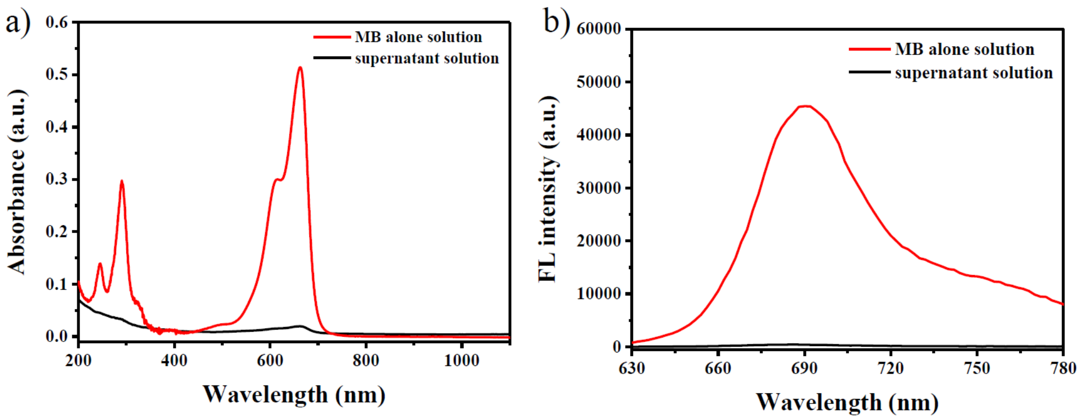

Figure S4. a) UV-visible and b) fluorescence measurements of the MB alone solution, referred as 100 % of concentration, and the supernatant of the Au@TNA@MB nanoparticles after 1 day of aging.
